# Joint Microbiota Suggests Articular Dysbiosis in Experimental Murine Spondyloarthritis and Histological Detection of Bacteria in Human SpA Joints

**DOI:** 10.1155/ijin/9982583

**Published:** 2025-11-11

**Authors:** Susana Aideé González-Chávez, María Fernanda Alvarado-Jáquez, Joan Sebastian Salas-Leiva, Jonathon E. Mohl, Eduardo Chaparro-Barrera, Rodrigo Prieto-Carrasco, Mario Loya-Rivera, César Pacheco-Silva, César Pacheco-Tena

**Affiliations:** ^1^PABIOM Laboratory, Faculty of Medicine and Biomedical Sciences, Autonomous University of Chihuahua, Chihuahua, Mexico; ^2^Department of Environment and Energy, Center for Research in Advanced Materials (CIMAV), Chihuahua, Mexico; ^3^Department of Mathematical Sciences, University of Texas at El Paso, El Paso, Texas, USA

**Keywords:** bacterial translocation, DBA/1 mice, dysbiosis, gut–joint axis, host–microbe interactions, IL-23 pathway, immune response, immunohistochemistry, short-read sequencing, tissue microbiota

## Abstract

**Background:**

Recent studies have provided evidence supporting the presence of a commensal joint microbiome; however, its role in the pathogenesis of spondyloarthritis (SpA) remains unclear. This study aimed to characterize the joint microbiome and assess its role in bacterial dissemination and systemic involvement.

**Methods:**

DBA/1 mice with spontaneous arthritis (SpAD) and healthy BALB/c mice, as well as biopsies from SpA patients, were analyzed by histology (Gram staining and IHC), short-read next-generation sequencing of the 16S rRNA gene amplicons, and transcriptomics. Shared bacterial species were evaluated across tissues, including the liver and heart, and the colocalization of bacterial and inflammatory markers was assessed using double indirect immunofluorescence (IIF).

**Results:**

Bacteria were detected in the joints of healthy and SpAD mice, with significantly greater abundance in the latter. Microbiome analysis revealed distinct bacterial communities, with genera, such as *Pelomonas* and *Aerococcus* uniquely identified in the joints of SpAD mice, indicating a state of dysbiosis. Several bacterial species, including *Prevotella* sp.*, Ruminococcus gnavus*, *Lactobacillus johnsonii*, and *Limosilactobacillus reuteri,* were detected in both the gut and joints of SpAD mice. Additionally, bacterial DNA from these taxa was also amplified from liver and heart tissues, indicating systemic dissemination. Transcriptomic analysis revealed dysregulated bacterial response pathways in SpAD joints, with an inflammatory profile distinct from that observed in gut tissues. Double IIF confirmed the colocalization of bacterial components with proinflammatory cytokines in joint cells. In human SpA biopsies, Gram staining and IHC also identified bacteria in sacroiliac and tarsal tissues.

**Conclusions:**

These findings confirm the presence of bacteria in the joints of healthy and SpAD mice, as well as SpA patients. The joint microbiome differs between healthy and diseased mice, contributing to inflammation through dysregulated bacterial responses. Additionally, the identification of shared bacterial species between the gut and joints, as well as their detection in the liver and heart, supports the hypothesis of bacterial dissemination consistent with translocation and systemic involvement.

## 1. Introduction

Spondyloarthritis (SpA) encompasses a group of chronic inflammatory diseases characterized by axial and peripheral joint involvement, enthesitis, and, in many cases, subclinical intestinal inflammation [1–3]. Recent evidence suggests that the gut may serve as both an immunological and microbial driver of joint pathology [4–6]. This has led to the conceptualization of a gut–joint axis, where intestinal dysbiosis may influence systemic inflammation and immune activation.

Multiple studies have documented alterations in the gut microbiome in patients with SpA, including changes in the abundance of specific bacterial taxa and a reduction in microbial diversity [7–9]. These alterations are thought to interact with genetic predisposition, particularly HLA-B27, to promote mucosal immune dysregulation [10–12]. In experimental models, such as HLA-B27 transgenic rats and SKG mice, gut inflammation and microbial imbalances precede or accompany joint manifestations [13, 14], further supporting the involvement of the gut in disease initiation. Notably, in the HLA-B27/β2-microglobulin transgenic rat, the absence of bacteria prevents the onset of arthritis [15].

The concept of a gut–joint axis has emerged to explain how intestinal alterations may directly contribute to joint inflammation in SpA. Clinical studies demonstrate that subclinical intestinal inflammation is frequent in SpA and correlates with disease activity, while histological and molecular analyses reveal impaired epithelial barrier integrity, characterized by increased zonulin and tight junction disruption [16–18]. Microbiome profiling further shows disease-specific dysbiosis, including reduced microbial diversity and enrichment of proinflammatory taxa, such as Dialister, which correlate with clinical severity [19, 20].

Mechanistically, bacterial epitopes cross-reacting with HLA-B27 provide evidence of molecular mimicry [7], and mucosal immunology studies reveal Type 17 innate-like responses and trafficking of gut-homing MAIT cells to inflamed synovial tissue, linking intestinal immune activation to joint pathology [21, 22]. Finally, the possibility of bacterial translocation from the gut to joints or bone marrow has been raised, suggesting that direct microbial dissemination may contribute to local dysbiosis and inflammation [23]. Collectively, these findings establish the gut–joint axis as a multidimensional model involving impaired barrier function, microbial dysbiosis, molecular mimicry, aberrant lymphocyte trafficking, and potential bacterial translocation, and are consistent with recent integrative reviews of this concept [24, 25].

This study explores an alternative hypothesis: that viable bacteria from the gut may translocate to the joint and other peripheral tissues, contributing directly to local dysbiosis and inflammation. While previous studies have reported microbial DNA in blood or joint samples [26–30], they do not confirm the viability or metabolic activity of these bacteria, nor do they evaluate their localization within joint structures.

Moreover, the concept of a resident joint microbiome has gained traction. Studies have described bacterial DNA and signatures in osteoarthritic cartilage and rheumatoid synovium [26, 31, 32], challenging the long-standing notion that the joint is a sterile compartment. Whether a similar microbiome exists in SpA-affected joints and whether it differs from that of healthy joints remain unexplored.

To address these gaps, this study characterizes the joint microbiome in a murine model of SpA and in human tissue samples, evaluates bacterial viability, identifies shared bacterial species between the gut and joint, and investigates their presence in distant organs, such as the liver and heart. This comprehensive approach aims to determine whether bacterial dissemination contributes to joint dysbiosis and inflammation in SpA.

## 2. Materials and Methods

### 2.1. Study Design

This experimental animal study aimed to confirm the presence of commensal and pathogenic microbiota in the joints of mice with and without arthritis, to explore the relationship between the joint and gut microbiomes, and to assess systemic dissemination of bacterial components. The study included DBA/1 mice, a murine model of SpA (SpAD), and healthy BALB/c mice used as controls. Due to the genetic predisposition of DBA/1 mice to develop joint inflammation, even in the absence of SpAD induction, they were not used as healthy controls. While DBA/1 mice naturally exhibit joint inflammation with age, BALB/c mice were chosen as controls because they remain free of inflammation throughout the study, providing a reliable nonarthritic baseline for microbiome comparisons.

Cross-sectional measurements were conducted posteuthanasia to assess (1) joint inflammation and remodeling via histopathology; (2) bacterial identification in knee joint of mice and human biopsies using Gram staining and immunodetection; (3) bacterial abundance and diversity in both knee and gut microbiomes through DNA-based cultivation-independent microbiome characterization; (4) shared bacterial species between knee and gut; (5) systemic dissemination by detecting bacterial DNA in liver and heart tissues through PCR; (6) transcriptomic expression profiles in the knee and gut of mice; (7) dysregulated host immune response to bacteria in SpAD joints; and (8) colocalization of bacterial components and inflammatory cytokines in the knee joint of diseased mice using double indirect immunofluorescence (IIF).

### 2.2. Animal Model

This study complied with the Official Mexican Standard NOM-062-ZOO-1999 for the care and use of laboratory animals. Approval was granted by the Ethics Committee and Institutional Animal Care and Use Committee (IACUC) at the Faculty of Medicine and Biomedical Sciences, Autonomous University of Chihuahua (ID number: CI-046-22).

A total of 30 male DBA/1 mice and 30 male BALB/c mice, all 10-week-olds, were included. SpAD was induced in DBA/1 mice according to the method described by Braem et al. [33], with the mice confined for 20 weeks in breeding cages, allowing 56 cm^2^ per mouse. BALB/c mice were maintained in the same type of cages but without space allocation restrictions per mouse. Both groups were housed in the same facility under controlled light (12-h light/dark cycles) and temperature conditions (23°C ± 2°C), with identical bedding, food, and water provided *ad libitum*. Despite the slight difference in space allocation, all other environmental conditions, including cage type, material, food, and water, were the same for both groups. Researchers and veterinarians monitored the mice throughout the study, and no adverse effects or behavioral issues were observed that required early termination of the experiment.

The sample size was calculated based on a comparison between two groups using quantitative data, with a Type I error of 5% and a power of 80%, and parameters derived from previous research on joint microbiomes in an OA murine model [32]. Specifically, the primary outcome for sample size estimation was the number of 16S rRNA gene amplicon reads from the OA study.

After 20 weeks, the mice were euthanized via isoflurane overdose, and samples were preserved for subsequent genetic and histological analysis.

### 2.3. Human Tissue Collection

Samples were collected from the tarsal bones of patients diagnosed with ankylosing tarsitis using bone-cutting forceps and dissection under local anesthesia with lidocaine [34]. Additionally, computed tomography–guided needle biopsies were obtained from the sacroiliac (SI) joint of patients with radiographic axial SpA, also with local anesthesia. Informed consent was obtained from all patients, and the procedures were approved by the Research and Ethics Committees of the General Hospital of Mexico “Dr. Eduardo Liceaga” and the Faculty of Medicine and Biomedical Sciences, Autonomous University of Chihuahua (ID number: FM-OEX-B-280/14 and CEI_EXP.102/13).

### 2.4. Histological Analysis of Inflammation and Bacterial Detection

Following euthanasia, the hind paws and knees of 10 mice from each group were dissected. One paw and knee from each mouse were fixed in 10% phosphate-buffered formalin and demineralized with 5% nitric acid; the other paw and knee were frozen in liquid nitrogen for transcriptomic analysis. Murine and human formalin-fixed tissues were dehydrated in graded ethanol and embedded in paraffin. Sections of 3 μm thickness were mounted on adhesive-coated glass slides and stained with hematoxylin & eosin (H&E). Digital images were captured using a camera (AmScope MU1803, Irvine, CA, USA) mounted on an optical microscope (AxioStar Plus, Carl Zeiss). In mouse joints, arthritis severity was assessed using a semiquantitative scale (0 = absent, 1 = mild, 2 = moderate, or 3 = severe) based on inflammatory infiltrate, enthesitis, cartilage damage, cartilage, bone neoformation, and ankylosis. Mean scores were calculated for each group.

Bacterial presence in the knee joints was evaluated in DBA/1-SpAD mice and BALB/c controls (10 per group) and human biopsies using Gram stains and immunohistochemistry (IHC). Modified Gram staining was performed according to Brown and Brenn [35], with picric acid used for background tissue staining. Optical microscopy at 100x magnification was employed to identify bacteria in knee cartilage, metaphysis, synovial membrane, bone, ligament, and bone marrow. Bacterial counts were conducted across 20 microscopic fields at 100x magnification, with the bacteria classified into Gram-positive or Gram-negative groups. These counts' means and standard errors (SEM) were calculated by joint structure and compared between groups.

The identification of bacterial components was performed using IHC with antibodies against lipid A (lipopolysaccharide, LPS; Invitrogen PA1-73178) and lipoteichoic acid (LTA, Invitrogen MA1-7402). Tissue sections were deparaffinized and rehydrated, and antigen retrieval was conducted with 0.05% trypsin. Tissues were permeabilized with 0.2% Triton X-100 (X100-100ML, Sigma Life Science), blocked with 10% bovine serum albumin, and endogenous peroxidase activity was removed using 3% hydrogen peroxide. Sections were incubated with primary antibodies at 37°C overnight (LPS: 1: 3000 and LTA: 1: 50), followed by the application of the corresponding biotin-streptavidin–conjugated secondary antibodies (Jackson ImmunoResearch Laboratories, Inc, PA, USA) and Pierce streptavidin horseradish peroxidase (Jackson ImmunoResearch Laboratories, Inc, PA, USA). Diaminobenzidine (DAB) (BSB0016, Bio SB, Inc, CA, USA) was used for chromogenic detection. Isotype controls were used by replacing the primary antibodies with isotype-matched control antibodies of the same species and concentration. Optical microscopy was conducted at 40x and 100x magnification, and images were captured using a digital camera (AmScope MU1803, Irvine, CA, USA).

Double IIF was used to identify the overlapping areas of expression of tumor necrosis factor alpha (TNF-α) and interleukin (IL)-23a with LPS and LTA. After deparaffinization and antigen retrieval, tissues were permeabilized with Triton X-100 and blocked with 5% donkey serum. The first primary antibodies (TNF-α, Invitrogen, PA5-120124; *IL-23a*, Bioss Antibodies, BS-1193R) were applied at 37°C overnight (TNF-α*:* 1:200 and *IL-23a*: 1:200), followed by incubation with AF488-labeled secondary antibodies (Donkey Anti-Mouse IgG or Donkey Anti-Rabbit IgG, Jackson ImmunoResearch Laboratories, Inc, PA, USA). A second blocking was performed before incubating with LPS (Invitrogen, PA1-73178) or LTA (Invitrogen, MA1-7402) antibodies. Cy5-labeled secondary antibodies were applied, and labeling was evaluated via epifluorescence microscopy (Zeiss Axio Imager A1). Images were acquired using a digital camera (AmScope MU1203-FL, Irvine, CA, USA).

### 2.5. Bacterial DNA Extraction, Amplification, and Quantification

All DNA isolation procedures were performed under sterile conditions in a biosafety cabinet. Instruments and consumables were sterilized and decontaminated by exposure to UV light for 30 min to minimize the risk of contamination [36, 37]. Negative controls were incorporated at each stage of the process, including DNA extraction, sacrifice, and dissection. To confirm the absence of contaminating bacterial DNA, PCR was performed using the primers PRK341F and PRK806R (Table 1). This PCR was applied to all reagents used in the extraction process (host depletion solution, selection buffer, DNA/RNA Shield, binding buffer, etc.), as well as to the liquid nitrogen and all other liquids used during the sacrifice and dissection procedures, to ensure no contamination from the reagents or materials.

Knee joints were harvested from 20 DBA/1 mice with SpAD and 20 healthy BALB/c mice. After euthanasia, the mice were immersed in absolute ethanol for 30 s to sterilize the surface. The skin was removed from the limbs, and the entire leg was dissected at the hip joint. The tissue was sterilized with 2% glutaraldehyde, rinsed with sterile 1X PBS, and processed within the biosafety cabinet. The knee joints were dissected by transecting the femur and tibia, collecting the joint with its cartilage, bone, ligaments, synovium, and bone marrow. The tissue samples were immediately frozen in liquid nitrogen and mechanically pulverized under the same conditions for DNA extraction.

Bacterial DNA was extracted from knee joint samples using the HostZERO Microbial DNA Kit (Zymo Research) according to the manufacturer's protocol and subsequently quantified using qPCR with the Femto Bacterial DNA Quantification Kit (Zymo Research), which provides high sensitivity and specificity for bacterial DNA detection, even in the presence of nonbacterial DNA. The procedure followed the manufacturer's instructions, and bacterial DNA content was quantified using the standard curve provided by the kit. Negative controls were included to ensure no contamination during the DNA isolation process.

After the dissection of the limbs, the liver and heart were harvested from DBA/1-SpAD mice. Additionally, stool samples were collected from the cecal region of both groups. The organs and stool were placed into sterile containers with DNA/RNA Shield (Zymo Research) for nucleic acid preservation and stored at −80°C until DNA extraction. Genomic DNA was extracted from liver and heart tissues using the E.Z.N.A. Tissue DNA Kit (Omega Bio-Tek), following the manufacturer's protocol. For stool samples, DNA was isolated using the Quick-DNA Fecal/Soil Microbe Mini-Prep Kit (Zymo Research), according to the manufacturer's instructions.

DNA extracted from liver and heart tissues was subjected to nested PCR to identify specific bacteria, including *Prevotella* spp., *Prevotella copri*, *Ruminococcus gnavus*, *Eisenbergiella tayi*, *Ventrimonas faecis*, *Lactobacillus johnsonii*, *Lactobacillus intestinalis*, and *Limosilactobacillus reuteri*. The process began with full-length 16S rRNA PCR amplification using the primers 16S (27F-1495R) (Table 1), followed by PCR amplification with species-specific primers (Table 1). HotStarTaq DNA Polymerase (Qiagen) was used for all reactions. PCR products were visualized on a 1% agarose gel to confirm amplification of the targeted bacterial species.

### 2.6. 16S rRNA Amplicon Sequencing and Microbiome Analysis

DNA from knee joints and stool samples from both groups of mice was quantified using a Qubit 4 Fluorometer (Thermo Fisher Scientific). For sequencing, equimolar pools were created from the DNA samples of both the knee joints and stool. Specifically, six pools of knee joint DNA were created from DBA/1-SpAD mice (*n* = 20), and four pools of knee DNA from BALB/c healthy mice (*n* = 20). Likewise, six pools of stool DNA were created from DBA/1-SpAD mice (*n* = 20), and four pools of stool DNA from BALB/c healthy mice (*n* = 20). This approach enabled the obtaining of sufficient DNA for sequencing, as individual DNA extractions from the knee joints were challenging due to low yields, especially from the BALB/c healthy mice. Although pooling DNA from multiple animals is generally not the preferred approach for microbiome analyses, this strategy was employed to ensure sufficient DNA for sequencing. The DNA from various animals within the same experimental group (DBA/1-SpAD or BALB/c healthy) was pooled while maintaining consistent treatment conditions for each group. This approach enabled group-level comparisons between joint and stool microbiomes within each experimental cohort.

To perform this DNA-based cultivation-independent microbiome characterization, sample preparation and DNA sequencing were conducted at the National Institute of Genomic Medicine (INMEGEN, Mexico), following standard Illumina amplicon protocols. The V3-V4 region of the 16S rRNA gene amplicon was amplified using the universal primers listed in Table 1, and sequencing was carried out on the Illumina MiniSeq platform under standard conditions. The raw reads generated from the 16S rRNA gene amplicon sequencing have been deposited in the NCBI Sequence Read Archive and are accessible under BioProject ID PRJNA1137856.

The Illumina reads were processed using QIIME2 (v.2022.8) [38]. In brief, the reads were imported and denoised using DADA2 [39]. Representative sequences were then assigned to a taxonomic group using the QIIME2 plugin greenegenes2 [40]. Taxonomic results were plotted as bar charts. The biom table was then exported for further analysis.

To estimate α diversity (Simpson and Shannon diversity indices and Chao-1 richness) and β diversity and to identify statistically significant differences, the “feature-table.biom” matrix was filtered by retaining only samples with a minimum of four counts, a prevalence of at least 20% across samples, and further refined using the interquartile range (IQR = 10%). The data were then normalized using the Cumulative Sum Scaling (CSS) method [41]. The taxa's relative abundances (A_rel_) were then used to describe community structure at the Class and Family levels, which were visualized using bar plots in the MicrobiomeAnalyst software [42]. A Kruskal–Wallis test was applied to assess differences in α diversity, with differences considered significant at *p* < 0.05 false discovery rate (FDR). Differences between groups were evaluated using the Bray–Curtis dissimilarity index and visualized through principal coordinate analysis (PCoA) plots. Significant variations in β diversity were tested using permutational multivariate analysis of variance (PERMANOVA) and analysis of similarities (ANOSIM) (*p* < 0.05-FDR).

Subsequently, pairwise comparisons between groups (single-factor comparisons) were performed using EdgeR (*p* < 0.05-FDR) to identify differential species [42]. In cases where no taxonomic classification was available at the studied rank, the classification of the following known higher rank was reported. Finally, a set analysis was performed to identify shared and nonshared genera between the knee and stool bacteria in the DBA/1-SpAD and BALB/c groups. For this purpose, Venn diagrams were generated, considering that a genus must be present in at least 20% of the samples (libraries) within each group, using the tool available at https://bioinformatics.psb.ugent.be/webtools/Venn/.

The dataset generated from 16S rRNA gene amplicon sequencing has been deposited in the NCBI database and is accessible under BioProject ID PRJNA1137856.

### 2.7. Transcriptome Analysis of Joint and Gut Tissues

The transcriptomes of knee joints from DBA/1-SpAD mice were compared with those of healthy BALB/c mice to identify the differentially expressed genes (DEGs) related to bacterial responses and defense mechanisms, with a focus on their potential roles in inflammation. Additionally, we compared the transcriptomes of knee joints and the ileocolic junction from DBA/1-SpAD mice to identify bacterial response and defense-related genes that were differentially expressed between these tissues.

The analysis focused on the biological processes *Response to bacterium* and *Defense response to bacterium*, given the aim of understanding how bacteria contribute to joint inflammation in the DBA/1-SpAD model. Although other processes were found to be deregulated in the transcriptomic analysis, these two were selected because they are directly related to immune responses to bacteria and inflammation in joint tissues. This selection was based on the hypothesis that DBA/1-SpAD mice may exhibit distinct immune responses to bacteria in the joints compared to healthy BALB/c mice. Processes associated with organ-specific functions, such as digestion or ossification, were intentionally excluded, as they fell outside the scope of this study. The primary focus was on the immune responses triggered by bacteria in both the joints and the gut, to explore the connection between gut and joint inflammation.

For transcriptomic analysis, knee joints and the ileocolic junction were collected from 10 mice per group (*n* = 10). Following dissection, the tissues were processed by removing the skin, hair, nails, and muscles, isolating the joint structures, which included bones, cartilage, and ligaments. The ileocolic junctions were dissected and rinsed with sterile 1X PBS to remove intestinal contents. Both tissues were immediately frozen in liquid nitrogen and pulverized. Total RNA was isolated using the RNeasy Tissue Mini Kit (Qiagen, MD, USA) following the manufacturer's protocol. RNA quality and integrity were confirmed using a Qubit 4 Fluorometer (Thermo Fisher Scientific). Equimolar RNA pools were prepared to analyze three DNA microarrays per group for tissue-specific studies.

Transcript expression analysis was performed using Mouse Clariom D microarrays (Applied Biosystems, USA). Biotinylated complementary DNAs were synthesized from 200 ng of total RNA according to the Affymetrix protocol in the GeneChip WT PLUS Reagent Kit Manual. After fragmentation, 5500 ng of fragmented and labeled ss-cDNA was hybridized for 18 h at 45°C on mouse GeneChips Clariom D Assay. GeneChips were washed and stained using the Affymetrix Fluidics Station 450 and then scanned on the Affymetrix GeneChip Scanner 3000. Data were analyzed using the Transcriptome Analysis Console (TAC) with Affymetrix default analysis settings and global scaling for normalization. The microarray dataset is registered in the Gene Expression Omnibus (GEO) under accession numbers GSE276030 and GSE278297.

DEGs were identified in TAC by comparing (1) transcriptomes from the knee joints of DBA/1-SpAD mice (experimental) and BALB/c mice (control) and (2) transcriptomes from the knee joints of DBA/1-SpAD mice (experiment) and the ileocolic junction of DBA/1-SpAD mice (control). DEGs were considered significant if they had an absolute log2 fold change (log2FC) ≥ 1.5 or ≤ −1.5 and *p* values ≤ 0.05-FDR.

For functional analysis, the list of DEGs was processed using the DAVID Bioinformatics Platform (https://david.ncifcrf.gov/) [43, 44] to identify genes associated with the Gene Ontology (GO) term *Response to bacterium* (GO: 0009617). Additionally, DEGs were analyzed using the STRING Platform v.12.0 (https://string-db.org/) [45] under stringent criteria (1% FDR) to identify genes related to *Defense response to bacterium* (GO: 0042742). Protein–protein interaction (PPI) networks for the identified DEGs were visualized on the STRING platform, and a subnetwork of genes involved in the *Inflammatory response* (GO: 0006954) was identified.

In the comparison between knee joints and the ileocolic junction in DBA/1-SpAD mice, the gut served as the control group to examine bacterial response mechanisms. Upregulated genes in the knee joints reflected the response to bacteria, while downregulated genes were associated with the gut response. PPI networks constructed from these gene lists revealed key clusters that clarify bacterial response mechanisms in both tissues.

### 2.8. Validation of Transcriptomic Findings by Reverse Transcription–Quantitative PCR (RT-qPCR)

RT-qPCR was performed to (1) validate the microarray results using the pyruvate dehydrogenase kinase 4 (*Pdk4*), collagen type X alpha chain 1 (*Col10a*), and osteocalcin (*Bglap2*); (2) assess the expression of cytokines relevant to SpA pathogenesis, including the *Tnfa*, *Il17f*, *Il23a*, and *Il33*; and (3) confirm 16S rRNA expression using primers for V3-V4 amplification (Table 1).

cDNA was synthesized from 1 μg of total RNA using the SensiFast cDNA Synthesis Kit (Meridian Bioscience) according to the manufacturer's instructions. For each gene, 3 μL of individual cDNA was used for qPCR using Maxima SYBR Green/ROX (Thermo Scientific) with gene-specific annealing temperatures as shown in Table 1. Primer specificity was verified by melting curve analysis. Each sample was run in duplicate, and relative quantification (RQ) was calculated using the ΔΔCt method (RQ = 2^−ΔΔCt^), with the ribosomal protein L (Rpl)13 serving as the reference gene [46].

### 2.9. Statistical Analysis

Data from histological analysis, Gram staining, and RT-qPCR were visualized and analyzed using BioRender (biorender.com). Statistical tests were applied according to data distribution: two-way ANOVA with Bonferroni's post hoc test for histology, two-way ANOVA with Tukey's post hoc test for Gram staining, and Welch's *t*-test for RT-qPCR. Statistical significance was set at a *p* value ≤ 0.05. Detailed statistical methods for microbiome and transcriptomic data are described in their respective section.

## 3. Results

### 3.1. Presence of Bacteria in Joints

#### 3.1.1. Abundance of Gram-Positive and Gram-Negative Bacteria in Joint Histological Sections of SpAD Mice

**Figure 1:**
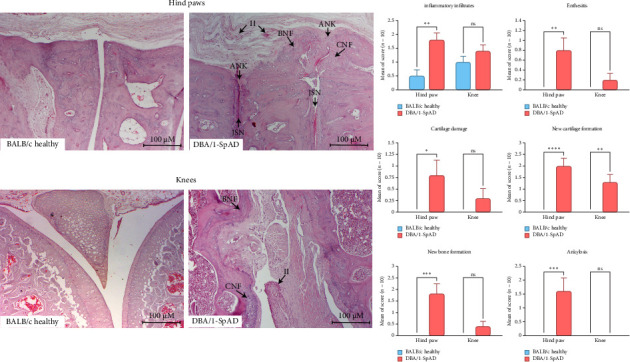
Histological evaluation of joint inflammation and remodeling in DBA/1-SpAD and BALB/c healthy mice. Representative H&E-stained sections of hind paws and knees from DBA/1-SpAD and BALB/c healthy mice. Histological features are indicated: II, inflammatory infiltrates; CNF, cartilage neoformation; BNF, bone neoformation; JSN, joint space narrowing; ANK, ankylosis. Arthritis severity was scored using a semiquantitative scale (0 = absent, 1 = mild, 2 = moderate, or 3 = severe), evaluating inflammatory infiltrate, enthesitis, cartilage damage, cartilage and bone neoformation, and ankylosis. Quantitative results (right panels) are presented as mean ± SEM (*n* = 10 per group). Statistical comparisons were performed using two-way ANOVA with Bonferroni's post hoc test. ^∗^*p* ≤ 0.05, ^∗∗^*p* ≤ 0.01, ^∗∗∗^*p* ≤ 0.001, ^∗∗∗∗^*p* ≤ 0.0001, ns = not significant.

Joint inflammation and remodeling in the hind paws and knees of DBA/1-SpAD mice were confirmed by H&E staining (Figure 1). Inflammatory infiltrates, enthesitis, cartilage damage, new cartilage formation, new bone formation, and ankylosis were higher in DBA/1-SpAD mice than in BALB/c healthy mice (*p* < 0.05). In arthritic mice, these parameters were more severe in the paws than in the knees. BALB/c mice showed no histological features of joint remodeling (Figure 1).

**Figure 2:**
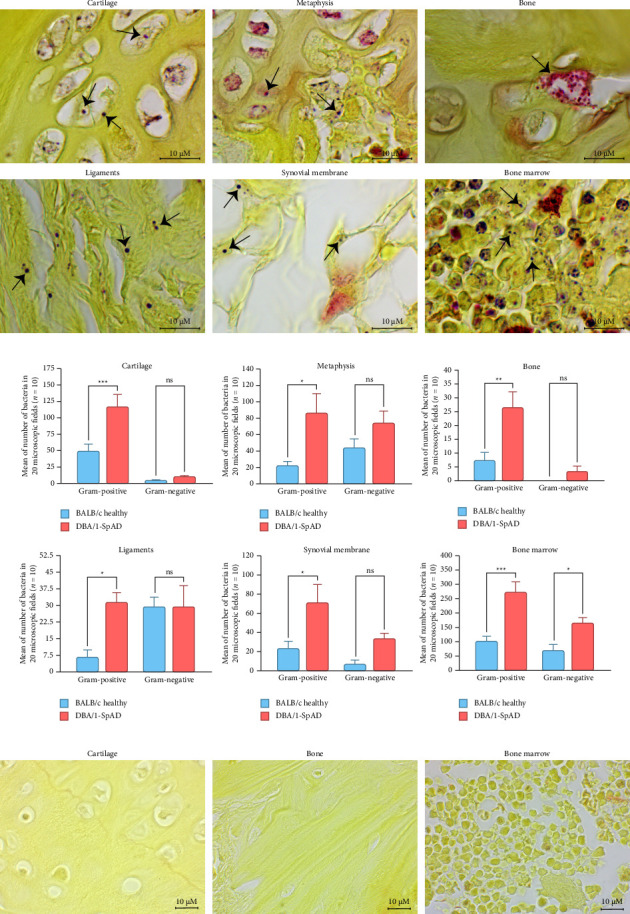
Identification of Gram-positive and Gram-negative bacteria by modified Gram stain in the knee joints of DBA/1-SpAD and BALB/c healthy mice. (a) Representative images of modified Gram stain in the knee joints in the different joint structures in diseased mice, including cartilage, metaphysis, bone, ligaments, synovial membrane, and bone marrow. Arrows indicate bacteria with Gram-positive (purple) or Gram-negative (pink/red) staining. (b) Quantification of Gram-positive or Gram-negative bacteria in each joint structure based on counts from 20 microscopic fields per sample at 100x magnification (10 mice per group). Data are expressed as mean ± SEM. Statistical comparisons were performed using two-way ANOVA with Tukey's post hoc test; ^∗^*p* ≤ 0.05, ^∗∗^*p* ≤ 0.01, ^∗∗∗^*p* ≤ 0.001. (c) Representative images of cartilage, bone, and bone marrow from healthy BALB/c mice, showing the absence of bacteria by modified Gram staining.

In the knees stained with the modified Gram stain for tissues, Gram-positive and Gram-negative bacteria were identified in joint samples from healthy (BALB/c) and diseased (SpAD) mice (Figures 2(a), 2(b), and 2(c)). A detailed quantification was performed to compare the bacterial abundance across different joint compartments and between groups. As shown in Figure 2(b), Gram-positive bacteria were significantly more abundant in all tissues analyzed (*p* < 0.05), with bone marrow exhibiting the highest bacterial load, followed by cartilage, metaphysis, and synovial membrane. When comparing groups, DBA/1-SpAD mice showed a significantly higher number of Gram-positive bacteria than BALB/c healthy mice in cartilage (*p* < 0.001), metaphysis (*p* < 0.05), bone (*p* < 0.01), ligaments (*p* < 0.05), synovial membrane (*p* < 0.05), and bone marrow (*p* < 0.001). Additionally, Gram-negative bacteria were significantly increased in SpAD mice only in bone marrow (*p* < 0.05), while no differences were observed in the remaining tissues. Representative negative control from healthy mice is shown in Figure 2(c).

**Figure 3:**
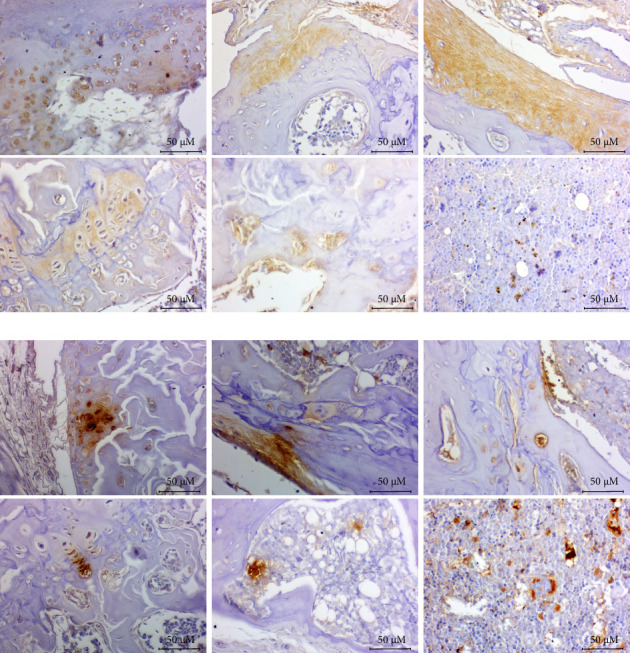
Presence of bacterial components in the joint structures. Representative images of bacterial components in the different joint structures by immunohistochemistry using antibodies for the lipopolysaccharide (LPS) component of Gram-negative bacteria (a) and lipoteichoic acid (LTA) component of Gram-positive bacteria (b). Optical microscopy was conducted at 40x magnification.

The presence of bacterial components in the different joint structures was confirmed by IHC using antibodies for LPS (Figure 3(a)) and LTA (Figure 3(b)). The staining with both antibodies was specific and had staining patterns located in defined and delimited areas. Bone marrow was the tissue with the highest detection of both antibodies.

#### 3.1.2. Detection of Bacteria in Human Biopsies From Patients With SpA

**Figure 4:**
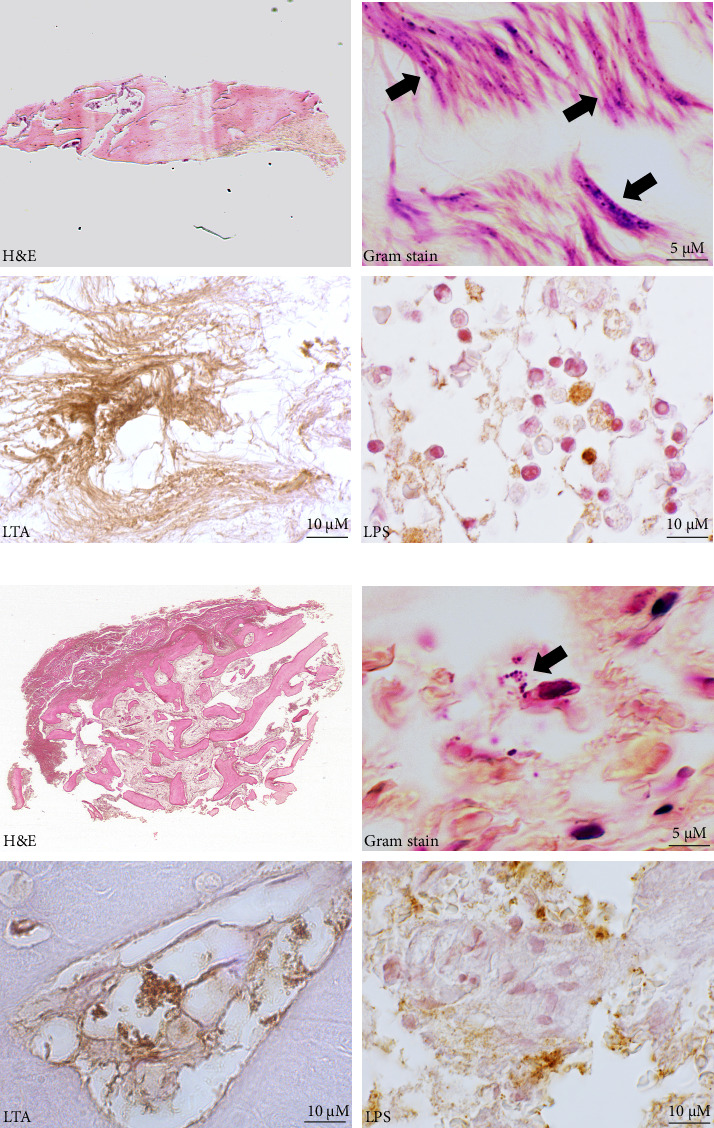
Detection of bacteria in human biopsies from patients with spondyloarthritis. Representative images of sacroiliac (a) and tarsus (b) joints in human biopsies, staining, and immunodetections. Bacteria and their components, lipopolysaccharide (LPS) and lipoteichoic acid (LTA), were identified by modified Gram stain and immunohistochemistry, respectively. Optical microscopy was conducted at 100x magnification.

In biopsies of SI joints and tarsal joints from patients with SpA, the presence of bacteria was confirmed using a modified Gram stain (Figure 4), primarily at the entheses and in the ligaments. Additionally, IHC identified bacterial components, LPS and LTA, in both the SI biopsies (Figure 4(a)) and the tarsals (Figure 4(b)) across different joint structures.

### 3.2. Characterization of Joint and Gut Microbiomes

#### 3.2.1. Bacterial DNA and RNA Content in the Joints of SpAD Mice

**Figure 5:**
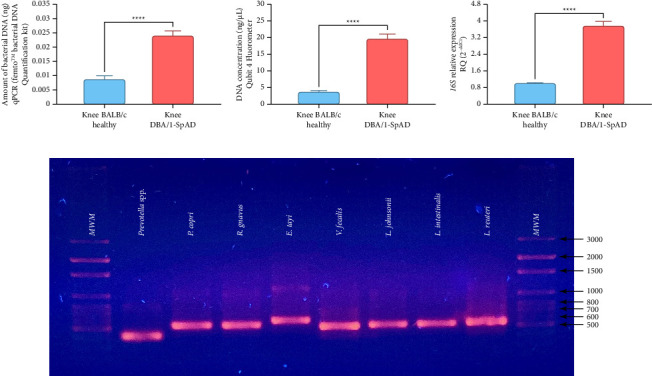
Quantification of bacterial DNA and RNA in the joints and detection of shared gut bacteria in the liver and heart of DBA/1-SpAD mice. (a) Bacterial DNA quantification in knee joints using Femto™ Bacterial DNA Quantification Kit. (b) The total DNA concentration in the knee joints was measured using the Qubit 4 Fluorometer. (c) Relative expression of 16S rRNA by RT-qPCR in knee joints. (d) Detection of shared gut–joint bacterial species (*Prevotella* spp., *P. copri*, *R. gnavus*, *E. tayi*, *V. faecalis*, *L. johnsonii*, *L. intestinalis*, and *L. reuteri*) in liver and heart tissues by nested PCR in DBA/1-SpAD mice. *n* = 20 mice per group. ^∗∗∗∗^*p* < 0.0001; Welch's *t*-test.

Bacterial DNA content in the knee joints of DBA/1-SpAD mice was found to be significantly higher than in healthy BALB/c mice. This was confirmed using both the qPCR Femto Bacterial DNA Quantification Kit (Zymo Research) (*p* < 0.0001, Figure 5(a)) and the Qubit 4 Fluorometer (Thermo Fisher Scientific) (*p* < 0.0001, Figure 5(b)). In addition, RT-qPCR analysis of the V3-V4 region of the 16S rRNA gene revealed higher expression levels of bacterial RNA in the joints of SpAD mice compared to healthy controls (*p* < 0.0001, Figure 5(c)).

#### 3.2.2. Amplicon Sequencing Confirms the Presence of a Joint Microbiome in SpAD and Healthy Mice

Before sequencing, rigorous quality control measures were implemented to exclude potential contamination. Negative controls were included throughout the DNA extraction, amplification, and sequencing processes, including extraction blanks (without tissue) and PCR controls (without DNA). In addition, all reagents used in the extraction and amplification procedures, including those for host depletion, selection buffer, DNA/RNA Shield, binding buffer, and liquid nitrogen, were tested for the absence of amplification. All negative controls yielded no amplification, ensuring that no contaminating DNA influenced the microbiome data. These controls confirm the integrity of the microbiome analysis, supporting the reliability of the findings from both knee and stool samples.

**Figure 6:**
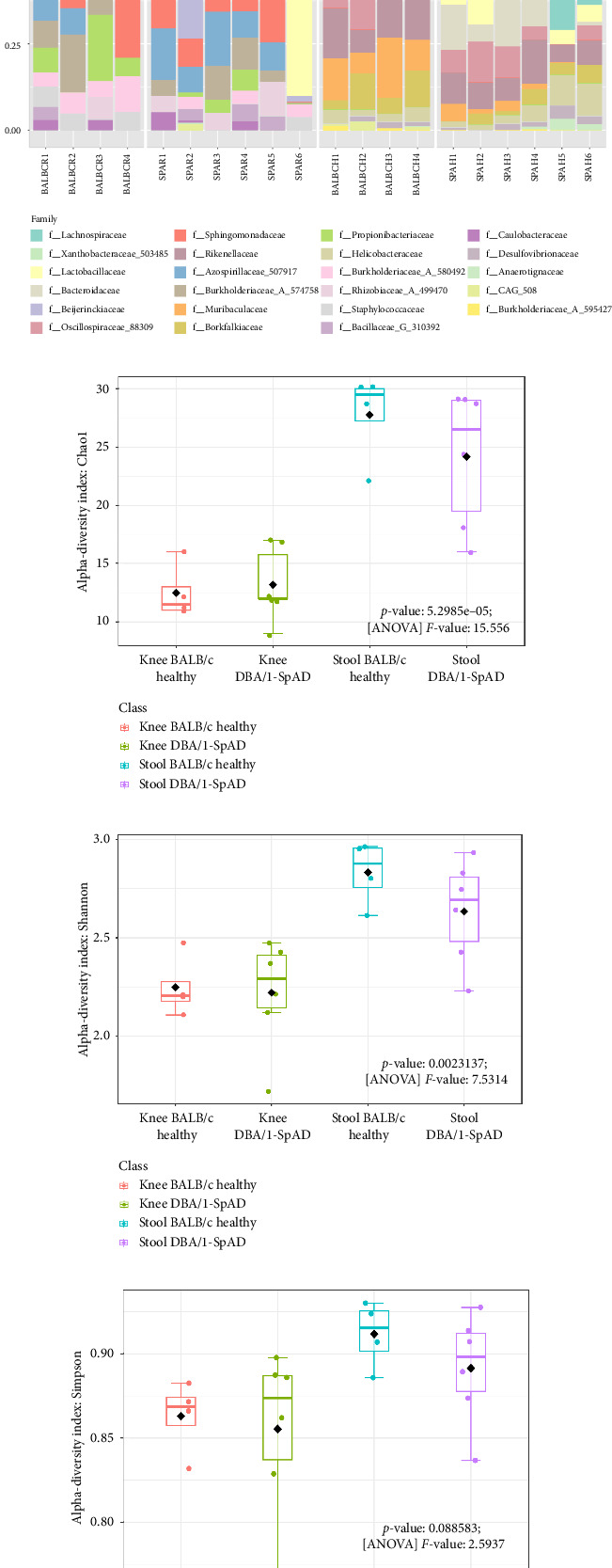
Structure and composition of knee and stool microbiomes. Relative abundances of the joint and gut microbiomes at class (a) and family (b) levels. Alpha diversity of the microbiomes is illustrated using the Chao-1 (c), Shannon (d), and Simpson (e) indices, with statistical analyses conducted using ANOVA. Beta diversity analysis (f) was performed using PERMANOVA. Specific *p* values and *F* values are indicated in the figure.

The analysis of the sequences from the knee and stool microbiomes showed distinct patterns in the relative abundances across all taxonomic levels. In the knee samples, the dominant classes were Alphaproteobacteria and Bacilli, whereas Clostridia and Bacteroidia were predominant in the stool samples (Figure 6(a)). At the order level, Rhizobiales and Lactobacillales were the most relatively abundant orders in the knee samples, while Bacteroidales and Lachnospirales dominated the stool samples. Similarly, at the family level, Xanthobacteraceae and Lactobacillaceae were prevalent in the knee, whereas Lachnospiraceae and Bacteroidaceae were most abundant in the stool (Figure 6(b)).

In the joint microbiome of both healthy and diseased mice, bacteria of the phylum Actinobacteriota were identified, which were absent from the gut microbiome. These included families, such as Xanthobacteraceae, Beijerinckiaceae, Sphingomonadaceae, Azospirillaceae, Burkholderiaceae, and Propionibacteriaceae (Figure 6(b)). Additionally, certain bacteria were exclusively found in stool samples, including members of Bacteroidia, Campylobacteria, and Desulfovibrionia. Some bacterial taxa were present in both tissues, including those from Bacilli, Clostridia, and Gammaproteobacteria (Figure 6(a)).

The alpha and beta diversity of the joint and gut microbiomes in both diseased and healthy mice are shown in Figure 4. The Chao-1 (Figure 6(c)), Shannon (Figure 6(d)(s)), and Simpson (Figure 6(e)) indices revealed significant differences in microbial diversity between study groups (*p* < 0.001-FDR), with stool samples exhibiting higher bacterial diversity compared to knee samples, as expected. Interestingly, bacterial diversity was reduced in the stool samples of diseased mice compared to healthy controls. Beta diversity analysis (Figure 6(f)) revealed a distinct clustering between joint and gut microbiomes and higher bacterial diversity in the knees of diseased mice compared to those of healthy mice (*p* < 0.001-FDR).

#### 3.2.3. Comparative Composition of Joint and Gut Microbiomes in DBA/1-SpAD and Control Mice

**Figure 7:**
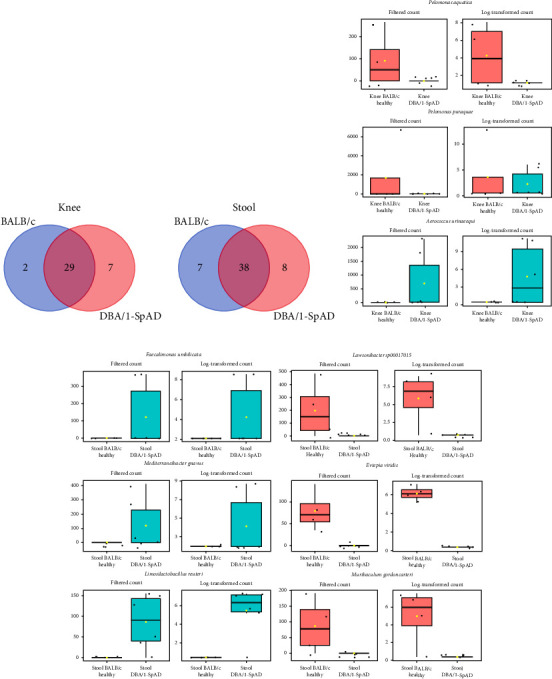
Comparison of joint and gut microbiomes between DBA/1-SpAD mice and healthy BALB/c mice. Venn diagrams of shared and nonshared genera between the BALB/c healthy and DBA/1-SpAD mice in the knees (a) and the stool (b) samples. Diagrams were generated considering a genus must be present in at least 20% of each group's samples (libraries). The lists of bacterial species represented in Venn diagrams are available in Supporting File 1. Pairwise comparisons between groups (single-factor comparisons) were performed using EdgeR (*p* < 0.05) to identify differential species between diseased and healthy mice in the joint (c) and stool (d) samples. Statistical significance was set at FDR ≤ 0.05.

All changes in bacterial levels reported below refer to relative abundances, given the compositional nature of 16S amplicon sequencing data. The comparison of joint microbiomes between DBA/1-SpAD mice and healthy BALB/c mice revealed 13 shared bacterial families, including Lactobacillaceae, Xanthobacteraceae, Beijerinckiaceae, Sphingomonadaceae, and Propionibacteriaceae. The bacterial species common to healthy and diseased joint microbiomes included *Bradyrhizobium ottawaense A 502985*, *Bradyrhizobium sp009781045*, and *Cutibacterium acnes*. The Venn diagram in Figure 7(a) shows that 29 bacterial species were found in the knees of both healthy and diseased mice. Additionally, it indicates that DBA/1-SpAD mice exhibited greater joint bacterial diversity compared to healthy mice, as shown in Supporting File 1.

Single-factor comparisons of joint bacterial species between diseased and healthy mice showed significant differences. *Pelomonas aquatica* and *Pelomonas puraquae* showed a lower relative abundance in SpAD mice (*p* < 0.05-FDR, Figure 7(c)), while *Aerococcus urinaeequi* showed a significantly higher relative abundance in the joint microbiome of diseased mice (*p* < 0.05-FDR, Figure 7(c)).

Similarly, the stool microbiomes of diseased and healthy mice also showed several shared bacteria, including those from the families Bacteroidaceae, Oscillospiraceae, Lachnospiraceae, Muribaculaceae, and Lactobacillaceae (Figure 7(b) and Supporting File 1). However, distinct species were also observed between the stools of healthy and diseased mice. *L. reuteri*, *Mediterraneibacter* (better known as *R. gnavus* [47]), and *Faecalimonas umbilicata* were relatively more abundant in SpAD mice (*p* < 0.01-FDR, Figure 7(d)), whereas *Lawsonibacter sp000177015*, *Evtepia viridis*, and *Muribaculum gordoncarteri* showed a lower relative abundance in SpAD mice (*p* < 0.01-FDR, Figure 7(d)).

#### 3.2.4. Shared Bacterial Profiles Between Joint and Gut Microbiomes

**Figure 8:**
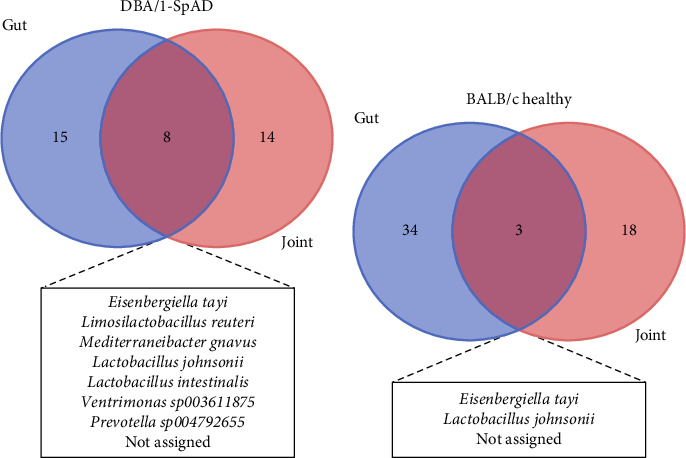
Shared bacterial profiles between joint and gut microbiomes in SpAD and healthy mice. Venn diagram of shared and nonshared genera between the knee and stool bacteria in the DBA/1-SpAD (a) and BALB/c healthy (b) mice. Diagrams were generated considering a genus must be present in at least 20% of each group's samples (libraries).

Joint and stool microbiomes were compared to find coincident bacteria between the gut and joint that could explain systemic dissemination and support the hypothesis of bacterial translocation. The core microbiomes of the joints and stool of DBA/1-SpAD mice and healthy BALB/c mice are shown in Figures 8(a) and 8(b), respectively.

Bacterial species in both the joint and stool samples were identified. In SpAD mice, *L*. *johnsonii*, *L*. *reuteri*, *E*. *tayi*, *Prevotella sp004792655*, *Ventrimonas sp003611875*, *L*. *intestinalis*, and *R*. *gnavus* were identified in both the knee and stool (Figure 8). In healthy mice, only *L. johnsonii* and *E*. *tayi* species were found in both tissues (Figure 8(b)). In healthy and diseased mice, shared bacteria were found between the knee and stool, whose sequences were not assigned to any species.

### 3.3. Detection of Bacterial DNA in Liver and Heart Tissues

To further investigate systemic dissemination of bacterial components, we examined liver and heart tissues from DBA/1-SpAD mice for the presence of bacterial DNA. Nested PCR was performed to identify specific bacterial species, including *P.* spp., *P*. *copri*, *R*. *gnavus*, *E*. *tayi*, *V*. *faecis*, *L*. *johnsonii*, *L*. *intestinalis*, and *L*. *reuteri*. Results showed successful amplification of DNA from these bacterial species in both liver and heart tissues of SpAD mice (Figure 5(d)). These findings provide indirect evidence consistent with bacterial translocation.

### 3.4. Transcriptomic and IIF Analysis of Inflammatory Responses

#### 3.4.1. Transcriptomic Profile of Joint and Gut Tissues of DBA/1-SpAD Mice

**Figure 9:**
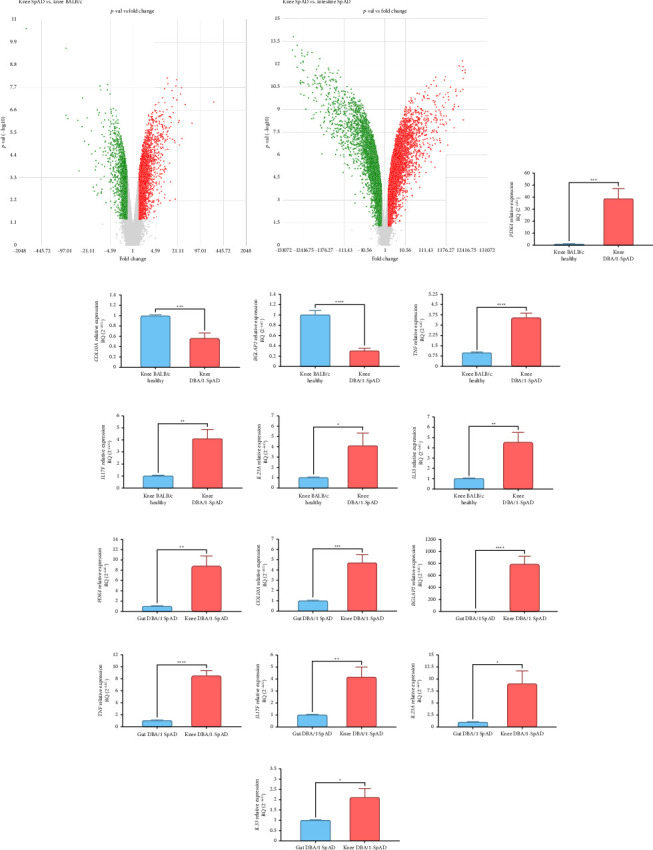
Differential transcriptomic profile in the joint and intestine of DBA/1 mice with SpAD. Volcano plots of differentially expressed genes (log2FC > 1.5 or < −1.5, *p* < 0.05) were generated from the transcriptomic comparison between the knee joints of diseased and healthy mice (a) and the knee joints and guts of diseased mice (b). Validation of microarrays by RT-qPCR included the genes *Pdk4* (c, j), *Col10a* (d, k), and *Bglap2* (e, l). Relative expression of *Tnf* (f, m), *Il17f* (g, n), *Il23a* (h, o), and *Il33* (i, p) between knee joints of diseased and healthy mice (f–i) and between knee joints and guts of diseased mice (j–p). *n* = 10 mice per group. Welch's *t*-test was used to compare measurements between groups. Statistical significance was set at a *p* value of ≤ 0.05. ^∗^*p* ≤ 0.05, ^∗∗^*p* ≤ 0.01, ^∗∗∗^*p* ≤ 0.001, ^∗∗∗∗^*p* ≤ 0.0001.

The comparison of joint transcriptomes between SpAD and healthy mice identified 6673 DEGs, comprising 4225 upregulated and 2448 downregulated genes (Figure 9(a)). In contrast, the analysis comparing joint and gut transcriptomes in SpAD mice revealed 14,866 DEGs, with 8033 genes upregulated and 6833 downregulated (Figure 9(b)).

The RNA expression levels observed in the microarray analyses were validated by RT-qPCR, confirming the overexpression of the *Pdk4* gene and the downexpression of *Col10a* and *Bglap2* in the joints of SpAD mice compared to healthy controls (Figures 9(c), 9(d), and 9(e)). These three genes were also validated as upregulated in the joint tissue compared to the gut of the same SpAD mice (Figures 9(j), 9(k), and 9(l)).

Furthermore, the knees of SpAD mice showed significantly elevated expression levels of the cytokines *Tnfa*, *Il17f*, *Il23a*, and *Il33* compared to healthy mice (Figures 9(f), 9(g), 9(h), and 9(i)). Additionally, these inflammatory cytokines were expressed at higher levels in the knees of SpAD mice than in the gut of the same diseased mice (Figures 9(m), 9(n), 9(o), and 9(p)).

#### 3.4.2. Bacterial Response Mechanisms in SpAD and Healthy Mice

**Figure 10:**
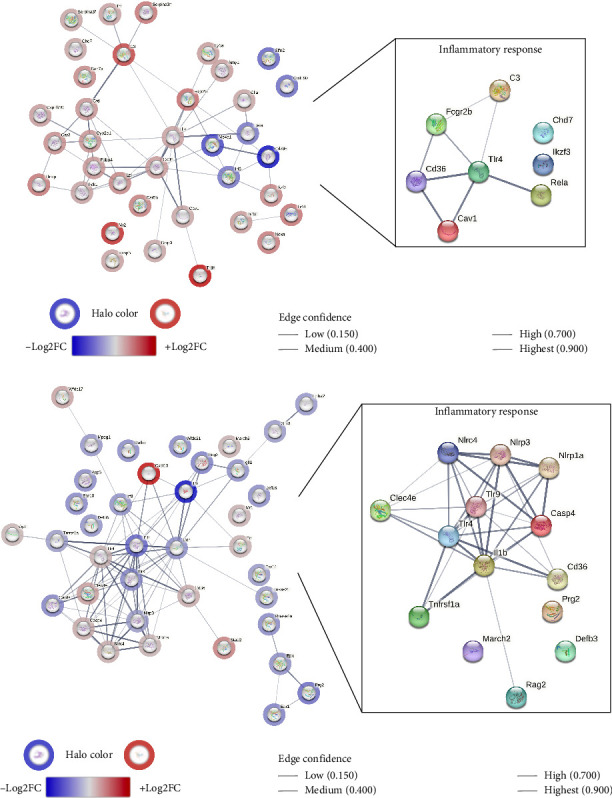
Differential bacterial response mechanisms in the joint of DBA/1-SpAD mice. Protein–protein interaction (PPI) networks of differentially expressed genes in the knee joints of diseased versus healthy mice, associated with the (a) “response to bacteria” (GO: 0009617) and (b) “defense response to bacteria” (GO: 0042742) processes. The color and intensity of the halos indicate the direction and magnitude of gene dysregulation (blue: downregulated; red: upregulated). The edges between nodes represent the confidence level of the PPI association. The boxes highlight the DEGs associated with the “inflammatory response” (GO: 0006954) process in these PPI networks.

The bioinformatic analysis of transcriptomes aimed to describe the mechanisms underlying bacterial responses in the joint tissues of SpAD and healthy mice. The findings revealed significant differences in the expression of genes related to bacterial response between the two groups. Specifically, the DEGs associated with the biological processes are depicted in Figure 10. In the process of *Response to bacterium* (Figure 10(a)), 44 genes were upregulated, while 10 were downregulated in the joints of SpAD mice. Conversely, in the *Defense response to bacterium* (Figure 10(b)), 25 genes were downregulated, and 13 were upregulated.

A subanalysis of genes involved in the *Inflammatory response* process (GO: 0006954) generated PPI networks, shown in the boxed sections of Figure 10. These networks suggest mechanisms linking inflammation to cellular responses to bacterial presence. As illustrated, the inflammatory response in the knee joints of SpAD mice was marked by the upregulation of genes, such as toll-like receptor (*Tlr*)*4*, the cluster of differentiation (*Cd*)*36*, complement component *C3*, caspase (*Casp*)*4*, and nucleotide-binding domain and leucine-rich repeat-containing (*NLR*) family proteins. In contrast, *I11b*, *Tlr9*, and *Rela* were downregulated.

#### 3.4.3. Comparative Bacterial Response Mechanisms in Joint and Gut Tissues

**Figure 11:**
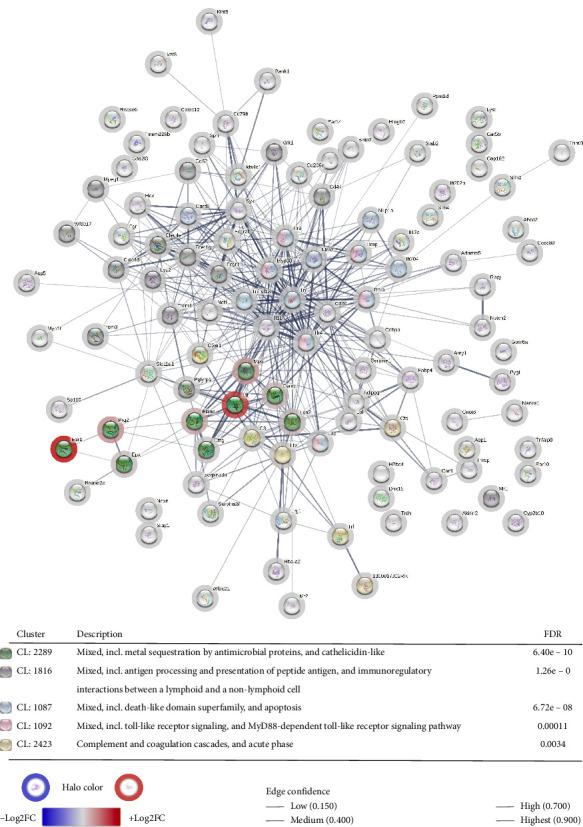
Bacterial response mechanisms in the joint of SpAD mice. Protein–protein interaction (PPI) network of the upregulated genes in the joint compared to the gut of DBA/1-SpAD mice, associated with the “response to bacteria” (GO: 0009617) and “defense response to bacteria” (GO: 0042742) processes. The color and intensity of the halos indicate the direction and magnitude of gene dysregulation (blue: downregulated; red: upregulated). The edges between nodes represent the confidence level of the PPI association. The table displays the clusters of processes and signaling pathways associated with this PPI network, highlighting the nodes belonging to each process with distinct colors.

**Figure 12:**
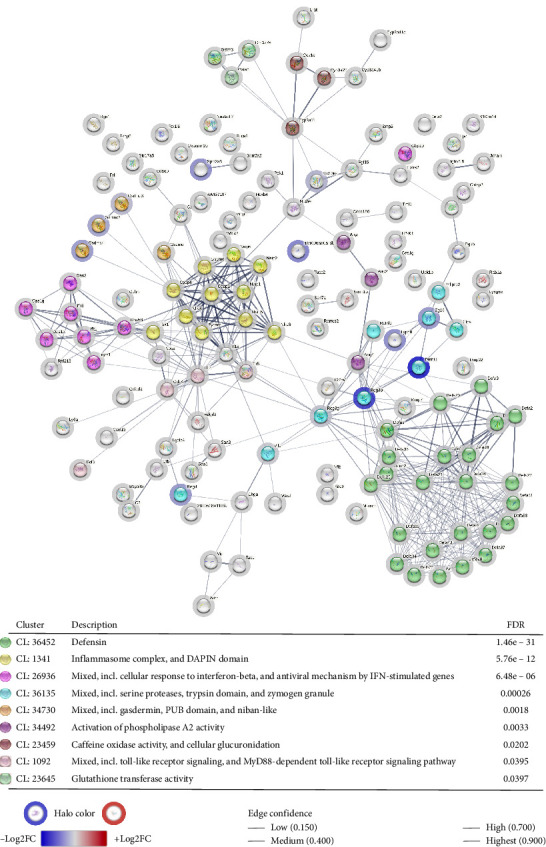
Bacterial response mechanisms in the gut of SpAD mice. Protein–protein interaction (PPI) network of the downregulated genes in the gut compared to the joint of DBA/1-SpAD mice, associated with the “response to bacteria” (GO: 0009617) and “defense response to bacteria” (GO: 0042742) processes. The color and intensity of the halos indicate the direction and magnitude of gene dysregulation (blue: downregulated; red: upregulated). The edges between nodes represent the confidence level of the PPI association. The table displays the clusters of processes and signaling pathways associated with this PPI network, highlighting the nodes belonging to each process with distinct colors.

Transcriptomic comparisons between joint and gut tissues in DBA/1-SpAD mice were conducted to delineate the differences in bacterial response mechanisms at these two sites. A PPI network was constructed using upregulated genes that represent the bacterial response in the joint (Figure 11). In contrast, a separate PPI network was created from downregulated genes to illustrate the bacterial response in the gut (Figure 12). Significant gene clusters were identified in both networks (indicated by colored nodes in the figures), with detailed descriptions provided in the accompanying tables.

The bacterial response gene clusters differed between the joint and the gut, except for *Mixed, including Tlr signaling and the MyD88-dependent Tlr signaling pathway* (CL:1092). In the knee joint, the response to bacteria was associated with clusters related to pathways, such as *Metal sequestration by antimicrobial proteins* (CL:2289), *Antigen processing and presentation of peptide antigen, and immunoregulatory interactions between a lymphoid and nonlymphoid cell* (CL:1816), *Death-like domain superfamily* (CL:1087), and *Complement and coagulation cascade* (CL:2423) (Figure 10). In contrast, bacterial response clusters in the gut were primarily associated with the *Defensin* pathway (CL:36452), *Inflammasome complex* (CL:1341), and *Cellular response to interferon-beta* (CL:26936) (Figure 12).

#### 3.4.4. Colocalization of Inflammatory Cytokine and Bacterial Components in SpAD Joints

The expression of TNF-α and IL-23a throughout joint structures was confirmed via IIF in the joints of SpAD mice. In the case of IL-23a, its highest expression was found in the metaphysis, while the bone marrow showed low protein expression levels. The expression of TNF-α was more ubiquitous, with stronger signals in the synovial membrane, ligaments, and entheses.

**Figure 13:**
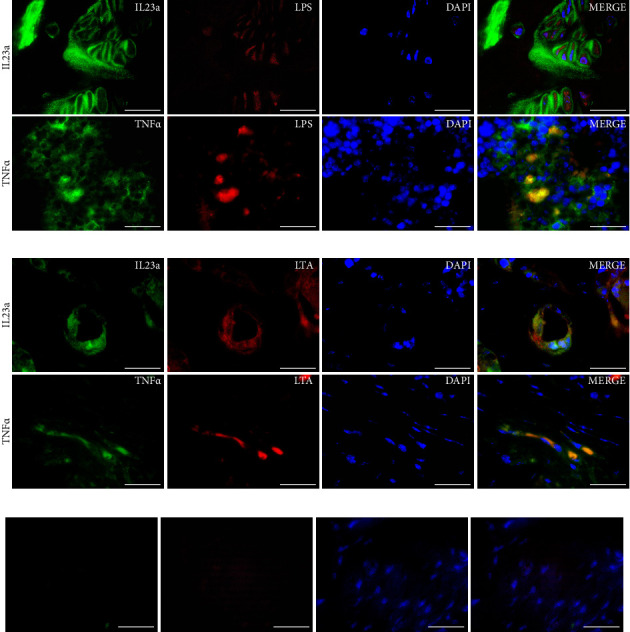
Colocalization of bacterial components and cytokines in the joints of DBA/1-SpAD mice. Representative images showing the colocalization of IL-23a and TNF-α with lipopolysaccharide (LPS), Gram-negative bacteria (a) and lipoteichoic acid (LTA), Gram-positive bacteria (b) through double immunofluorescence in the joint structures of DBA/1-SpAD mice. DAPI was used to stain nuclei. (c) Isotype controls for primary antibodies confirmed staining specificity. Scale bar: 20 μm.

Double IIF of inflammatory cytokines, LPS, and LTA demonstrated that the histological localization of bacterial components overlaps with areas where inflammatory cytokines are expressed. As shown in Figure 13(a), the colocalization of LPS and IL-23a in the metaphysis highlights areas where LPS is present, surrounded by the cytokine in the chondrocytes. Additionally, the colocalization of LPS and TNF-α in the bone marrow reveals convergence regions.  Figure 13(b) illustrates how LTA overlaps with IL-23a in a bony region, while it is present with TNF-α at the enthesis. To validate the specificity of the immunofluorescent staining, isotype controls were included and showed no detectable signal (Figure 13(c)), confirming the specificity of the observed colocalization patterns.

## 4. Discussion

This study confirms the presence of commensal and pathological microbiomes associated with SpA and explores their role in the gut–joint axis. Bacteria were identified in the joints of both diseased and healthy mice, with significant differences in abundance and diversity. Gram-positive and Gram-negative bacteria were also detected in biopsies from patients with SpA. The presence of 16S rRNA transcripts suggests that some of these bacteria are viable and metabolically active. In SpAD mice, bacterial components colocalized with proinflammatory cytokines in inflamed joints, supporting their contribution to local inflammation. Shared bacterial species were detected between joints and gut in diseased mice, and the same taxa were also identified in liver and heart tissues, indicating systemic dissemination consistent with bacterial translocation. Together, these findings support the role of joint dysbiosis in the pathogenesis of SpA.

The concept of a joint microbiome, once controversial, is gaining support [48–54]. It has been described in synovial tissue from patients with rheumatoid arthritis (RA) [55–58], and confirmed in murine and human OA models [32, 59]. To our knowledge, this is the first study to demonstrate the coexistence of commensal and pathological microbiomes in the joints of a murine SpA model.

Bacterial presence was confirmed by modified Gram staining in both mouse joints and biopsies from patients with SpA. Although this technique has low sensitivity for septic arthritis [60–62], it provided the first visual evidence of bacteria in joint tissues. These findings were validated by LPS and LTA immunodetection and supported by sequencing data. Notably, bacteria followed consistent localization patterns in hyaline cartilage and the metaphysis, which differ in oxygen tension and metabolism [63, 64], suggesting the coexistence of anaerobic and aerobic bacteria in the joint microenvironment.

In mice, Gram staining revealed the highest bacterial load in the bone marrow and the lowest in the bone tissue. As bone marrow is a key inflammatory site in SpA, and bone marrow edema correlates with osteitis and clinical symptoms [65, 66], the presence of bacteria may contribute to inflammatory changes, such as fat metaplasia and sclerosis [67], supporting a role for bacteria in driving bone marrow pathology.

Based on these findings, the analysis was extended to human biopsies. Bacteria were detected in SI and tarsal joints from patients with SpA, confirmed by Gram staining and LPS/LTA immunodetection. Bacterial structures were observed in both the bone marrow and entheses, regions known for intense inflammation in SpA [68], supporting their potential involvement in the disease process.

Microbiome analysis confirmed the presence of bacteria in the joints of both healthy and SpAD mice. Dominant bacterial classes included Alphaproteobacteria and Bacilli, previously reported in joint tissues of OA patients and murine models [32, 59]. Notably, the phylum Actinobacteriota was detected in joints but not in gut samples, despite its recognized presence in the joints of RA and OA [54, 58, 59, 69] and its increased abundance in the gut microbiome of SpA patients [70].

Compared to healthy controls, SpAD mice exhibited significantly higher bacterial abundance and diversity across all joint compartments, consistent with intra-articular dysbiosis. These findings align with previous studies on OA and RA [32, 54, 58, 59, 71] and support the hypothesis that dysbiosis may contribute to joint inflammation in SpA. Some species, such as *C. acnes*, were shared by both groups and are frequently isolated from native and prosthetic joints [69, 71–73]. Reports of its presence in deep tissues without surgical access [74–77] suggest hematogenous dissemination. In contrast, *P*. *aquatica*, *P*. *puraquae,* and *A*. *urinaeequi* showed group-specific abundance patterns and have been linked to joint or musculoskeletal infections [78–80].

Several bacterial species were shared between the gut and joint microbiomes of SpAD mice, including *E*. *tayi* and *L*. *johnsonii*, while others, such as *L*. *reuteri*, *Prevotella*, *Ventrimonas*, *L*. *intestinalis*, and *R*. *gnavus*, were exclusive to the SpAD group. *R*. *gnavus* and *Prevotella* are consistently enriched in RA, lupus, IBD, and SpA [19, 81–86], and are associated with severe disease and immune imbalance. The identification of potentially active *R. gnavus* in the joints of SpAD mice is a novel finding that further supports a link between gut-derived bacteria and joint inflammation.

The DBA/1-SpAD model exhibited inflammation and increased permeability [87], likely facilitating the systemic dissemination of bacteria or bacterial components, which is compatible with translocation to the joint. While translocation has been proposed in OA [6, 31], it remains underexplored in SpA. However, studies in other models have shown that bacterial migration from the gut can drive systemic autoimmunity [88] and contribute to tumor colonization [89]. This study contributes to the existing evidence by demonstrating the presence of shared bacterial species in the gut, joints, liver, and heart of SpAD mice, suggesting systemic dissemination. Similar findings of bacterial DNA in the blood of patients and murine models with SpA [29, 30, 90, 91] support the hypothesis that microbial translocation contributes to systemic immune dysregulation and may represent a target for future biomarkers of disease progression.

These findings support a novel hypothesis within the gut–joint axis: that viable bacteria may disseminate from the gut and reach the joint, in a process consistent with bacterial translocation, thereby contributing directly to intra-articular dysbiosis and inflammation. This contrasts with existing models of SpA pathogenesis, which focus on arthritogenic peptides, aberrant lymphocyte trafficking, or gut dysbiosis that affect distant joints via indirect mechanisms [24, 92, 93]. By demonstrating the presence of metabolically active bacteria in joint tissues and their detection in distant sites, such as the liver and heart, this study provides evidence of bacterial dissemination to peripheral tissues, supporting the hypothesis of translocation from the gut to the joint.

To explore how bacteria may contribute to local inflammation, transcriptomic analysis was performed on joint tissues from SpAD and healthy mice. DEGs were enriched in two bacterial response categories: *Response to bacterium* (GO: 0009617), which includes any change in cellular activity triggered by bacterial stimuli, and *Defense response to bacterium* (GO: 0042742), associated with protective immune mechanisms [94, 95]. The former encompasses responses to both commensals and pathogens, while the latter reflects classical antibacterial defense.

In the *Response to bacterium* category, most DEGs were upregulated, suggesting activation of both innate and adaptive immunity. Conversely, in the *Defense response to bacterium* category, most DEGs were downregulated, indicating that the inflammatory response may proceed in the absence of classical antimicrobial activation. This dual behavior suggests that bacteria in the joint trigger immune activation while evading elimination, supporting a model of chronic dysbiosis rather than overt infection.

Bacterial presence was further confirmed by the colocalization of LPS and LTA with TNF-α and IL-23a via double IIF, in line with the transcriptomic upregulation of *Tnfa*, *Il17f*, *Il23a*, and *Il33*. This pattern resembles findings in other tissues, where bacterial dysbiosis drives inflammation without infection, such as in the central nervous system [96], lungs [97], bladder [98], and gut [99]. Moreover, the role of bacteria as direct triggers of tissue inflammation is well supported in the literature [100–103], reinforcing the hypothesis that joint inflammation in SpA may result from persistent bacterial presence. If viable bacteria contribute to the inflammatory environment, this would mark a paradigm shift in understanding SpA pathogenesis, moving beyond antigenic mimicry and passive dissemination toward a model of chronic microbial stimulation, as supported by the transcriptomic signature.

Transcriptomic analysis also revealed tissue-specific immune signatures. In the gut, the upregulation of defensins, particularly alpha-defensins from Paneth cells, suggests a compensatory response to dysbiosis, consistent with previous reports linking reduced defensin expression to intestinal inflammation and impaired microbial control [104–106]. Concurrent activation of the inflammasome-related genes and gasdermin-mediated pyroptosis [107–112] supports a localized antibacterial program driven by innate immunity. In contrast, the transcriptomic profile of the joint exhibited a markedly different pattern. An upregulation of genes encoding calprotectin and other metal-sequestering antimicrobial proteins was observed [113, 114], along with enrichment of pathways associated with complement activation, coagulation cascades, and acute-phase responses. Pathways related to peptide antigen presentation and death-like domain, previously implicated in arthritis [115–117], were also upregulated, reflecting the combined activation of innate and adaptive immunity in response to bacterial presence.

These results raise important questions about the definition of infection in SpA. While destructive infection is typically absent, the persistence of viable bacteria in joint tissues may represent a form of low-grade colonization or symbiosis that contributes to chronic inflammation. This phenomenon, observed in OA as well [6, 31, 32, 48], challenges traditional distinctions between infection and sterile inflammation, highlighting the need to reconsider the role of microbial persistence in joint disease.

While this study provides novel insights into the role of viable bacteria in joint inflammation, several limitations must be considered. First, Gram staining is primarily used in bacterial cultures and is not typically applied to tissue sections, particularly complex murine or human samples. This technique may lead to misinterpretation due to overlapping structures resembling bacteria. To address this, only well-defined bacterial forms were considered, and the findings were validated using IHC with antibodies against prokaryotic components.

Regarding microbial characterization, 16S rRNA amplicon sequencing was employed, which limits taxonomic resolution and may overlook specific microbial taxa. Due to low DNA yield, particularly in BALB/c mice, pooling of samples was necessary, which precludes individual-level analysis. Despite this, the pooled data provided sufficient depth to identify distinct bacterial communities and dysbiosis patterns between strains. Moreover, while BALB/c mice were chosen as controls because they remain free of spontaneous joint inflammation and therefore provide a reliable nonarthritic baseline, we acknowledge that their different genetic background compared to DBA/1 mice may influence microbial communities. This choice represents a methodological limitation, as it precludes direct strain-to-strain comparisons; however, it allowed us to contrast arthritic versus nonarthritic conditions in a reproducible way.

The extrapolation of murine data to humans should be done cautiously, as interspecies differences in the gut and potentially joint microbiomes exist. Nonetheless, many bacterial taxa identified in DBA/1-SpAD mice, such as *P. copri* and *R. gnavus*, have been previously associated with human inflammatory joint diseases.

In addition, the cross-sectional design represents an important limitation, as it restricts our ability to determine the temporal sequence between microbial alterations and disease progression. While our findings demonstrate the presence of viable bacteria in inflamed joints, they do not establish whether microbial dissemination precedes or follows the onset of inflammation. This was not within the objectives or scope of this study, which aimed to provide a proof-of-concept characterization at the time of disease manifestation. Serial sampling in mice is inherently unfeasible because joint harvesting requires euthanasia; however, future studies could employ staggered-sacrifice cohorts to reconstruct temporal dynamics across disease stages. In the human setting, longitudinal clinical studies with repeated microbiome sampling will be essential to validate whether joint microbial changes anticipate disease exacerbations or reflect ongoing inflammation.

Another limitation is that the human biopsy samples used were archival tissues without detailed clinical information. Consequently, we were unable to assess correlations between bacterial load or the presence of specific bacterial taxa and disease severity. Additionally, microbiome analysis was not possible due to the use of paraffin embedding. Despite these limitations, the detection of bacteria in SI and tarsal tissues from SpA patients represents a significant and novel observation that complements our experimental findings.

## 5. Conclusion

This study provides evidence of bacteria in the joints of healthy and SpA mice, as well as in SI and tarsal tissues from patients with SpA. SpAD mice exhibited intra-articular dysbiosis, characterized by bacterial species shared between the gut and joints, as well as in distant organs, consistent with systemic dissemination. These findings support the hypothesis that microbial migration and dysbiosis may contribute to joint inflammation in SpA, while emphasizing the need for further studies to establish causality and explore their potential as diagnostic or therapeutic targets.

## Figures and Tables

**Table 1:** Genes evaluated by PCR and qPCR.

Gene	Description	Primers (5′-3′)	T°
16S	16S (27F-1495R)	Fw 5′-GAGAGTTTGATCCTGGCTCAG	60°C
		Rev 5′-CTACGGCTACCTTGTTACGA	

16S	16S V3-V4 (PRK341F- PRK806R)	Fw 5′-CCTACGGGRBGCASCAG	53°C
		Rev 5′-GGACTACYVGGGTATCTAAT	

16S	16S V3-V4 (illumina)	Fw 5′-TCGTCGGCAGCGTCAGATGTGTATAAGAGACAGCCTACGGGNGGCWGCAG	55°C
		Rev 5′-GTCTCGTGGGCTCGGAGATGTGTATAAGAGACAGGACTACHVGGGTATCTAATCC	

Pdk4	Pyruvate dehydrogenase kinase 4	Fw 5′-CTTGTGGCTGAGCATTGCAG	63°C
		Rev 5′-GCCTCACGCACATCACTAGT	

Col10a	Collagen type X alpha chain 1	Fw 5′-TTCTGCTGCTAATGTTCTTGACC	63°C
		Rev 5′-GGGATGAAGTATTGTGTCTTGGG	

Bglap2	Osteocalcin	Fw 5′-CTGACCTCACAGATCCCAAGC	54°C
		Rev 5′-TGGTCTGATAGCTCGTCACAAG	

TNFa	Tumor necrosis factor alpha	Fw 5′-CCCTCACACTCAGATCATCTTCT	60°C
		Rev 5′-GCTACGACGTGGGCTACAG	

Il17f	Interleukin-17f	Fw 5′-TGCTACTGTTGATGTTGGGAC	63°C
		Rev 5′-AATGCCCTGGTTTTGGTTGAA	

Il23a	Interleukin-23a	Fw 5′-ATGCTGGATTGCAGAGCAGTA	59°C
		Rev 5′-ACGGGGCACATTATTTTTAGTCT	

Il33	Interleukin-33	Fw 5′-TCCAACTCCAAGATTTCCCCG	55°C
		Rev 5′-CATGCAGTAGACATGGCAGAA	

Rpl13	Ribosomal protein L 13	Fw 5′-AGCCTACCAGAAAGTTTGCTTAC	60°C
		Rev 5′-GCTTCTTCTTCCGATAGTGCATC	

*Prevotella* spp.	*Prevotella* spp.	Fw 5′-GAGAGCCTGAACCAGCCAAG	60°C
		Rev 5′-CCTGTTCGATACCCGCACTT	

*P. copri*	*P. copri*	Fw 5′-GAGGAAGGTCCCCCACATTG	55°C
		Rev 5′-GCATCCATCGTTTACCGTGC	

*R. gnavus*	*R. gnavus*	Fw 5′-GACGATCAGTAGCCGACCTG	60°C
		Rev 5′-GTTTACGGCGTGGACTACCA	

*E. tayi*	*E. tayi*	Fw 5′-GCCACATTGGGACTGAGACA	60°C
		Rev 5′-TTCTTGCGAACGTACTCCCC	

*V. fecalis*	*V. fecalis*	Fw 5′-CGGCGAGACAAGTCTGAAGT	60°C
		Rev 5′-CCCAACATCTCACGACACGA	

*L. johnsonii*	*L. johnsonii*	Fw 5′-GCCACATTGGGACTGAGACA	60°C
		Rev 5′-AGCACTCATCGTTTACGGCA	

*L. intestinalis*	*L. intestinalis*	Fw 5′-AACTGGCCCCTATTTGACGG	55°C
		Rev 5′-CCTGGTAAGGTTCTTCGCGT	

*L. reuteri*	*L. reuteri*	Fw 5′-AACTCCATGTGTAGCGGTGG	60°C
		Rev 5′-GATGATCTGACGTCGTCCCC	

## Data Availability

The data that support the findings of this study are openly available in the NCBI Sequence Read Archive (SRA) at https://www.ncbi.nlm.nih.gov/sra/?term=PRJNA1137856, under reference number PRJNA1137856. The microarray datasets supporting this article are also available in the Gene Expression Omnibus (GEO) under accession numbers GSE276030 and GSE278297.

## Supporting Information

Supporting File 1 lists the shared and distinct bacterial species identified in knee and stool samples from healthy BALB/c and DBA/1-SpAD mice. This dataset complements the main text by detailing the microbial taxa detected in both intestinal and articular compartments, supporting the evidence of joint dysbiosis in experimental SpA.
