# Supplementary material for: Joint Microbiota Suggests Articular Dysbiosis in Experimental Murine Spondyloarthritis and Histological Detection of Bacteria in Human SpA Joints
Source: Int J Inflam. 2025 Nov 11;2025:9982583. doi: 10.1155/ijin/9982583 (PMC12626697; doi:10.1155/ijin/9982583)
Supplement: Supporting Information — Additional supporting information can be found online in the Supporting Information section. [file 9982583.f1.pdf]

Supplementary File 1. Shared and distinct bacterial species between healthy BALB/c mice and DBA/1-SpAD mice in knees and stool samples.

A. KNEES

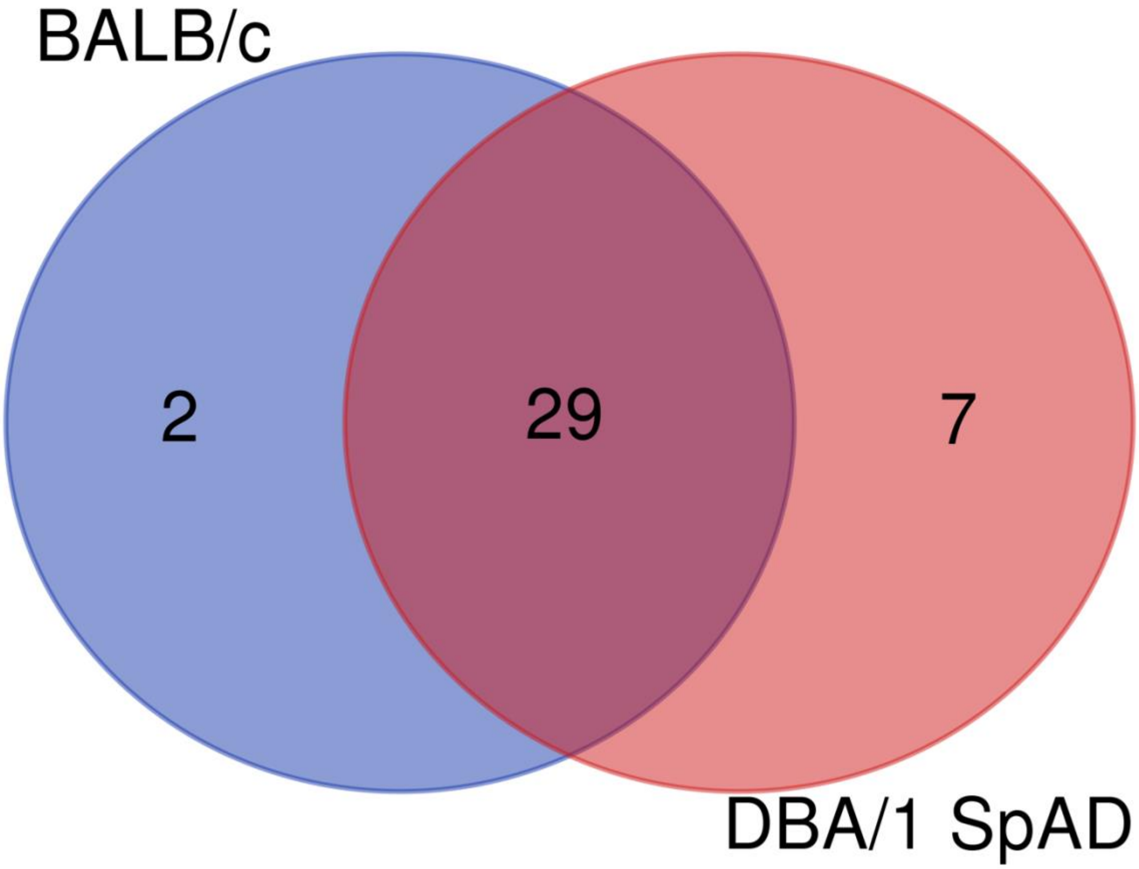

Group	Total	Bacterial spcecies
BALB/c healthy<br><br>DBA/1 SpAD	29	Massilia_suwonensis Methylobacterium_fujisawaense Brucella_melitensis Paraburkholderia_fungorum Paracoccus_aestuarii Eisenbergiella_tayi Methylobacterium_persicinum Staphylococcus_epidermidis_318824 Cutibacterium_acnes Staphylococcus_epidermidis_319181 Ralstonia_pickettii_B Micrococcus_luteus Pelomonas_puraquae Bosea_vaviloviae_B_503600 Staphylococcus_coagulans Not_Assigned Sphingomonas_L_486704_kyeonggiensis Bradyrhizobium_sp009781045 Azospirillum_thiophilum Massilia_tieshanensis Methylocella_palustris Lactobacillus_johnsonii Rubellimicrobium_mesophilum Staphylococcus_hominis Priestia_megaterium Acidovorax_A_kalamii Bradyrhizobium_ottawaense_A_502985 Comamonas_F_589250_antarcticus Sphingobium_A_485959_scionense
BALB/c healthy	2	Pelomonas_aquatica Skermanella_aerolata
DBA/1 SpAD	7	Acinetobacter_radioresistens Limosilactobacillus_reuteri Aerococcus_urinaeequi Azonexus_agitatus Bradyrhizobium_guangdongense Jeotgalicoccus_A_310962_aerolatus UBA5216_sp902825845

B. STOOL

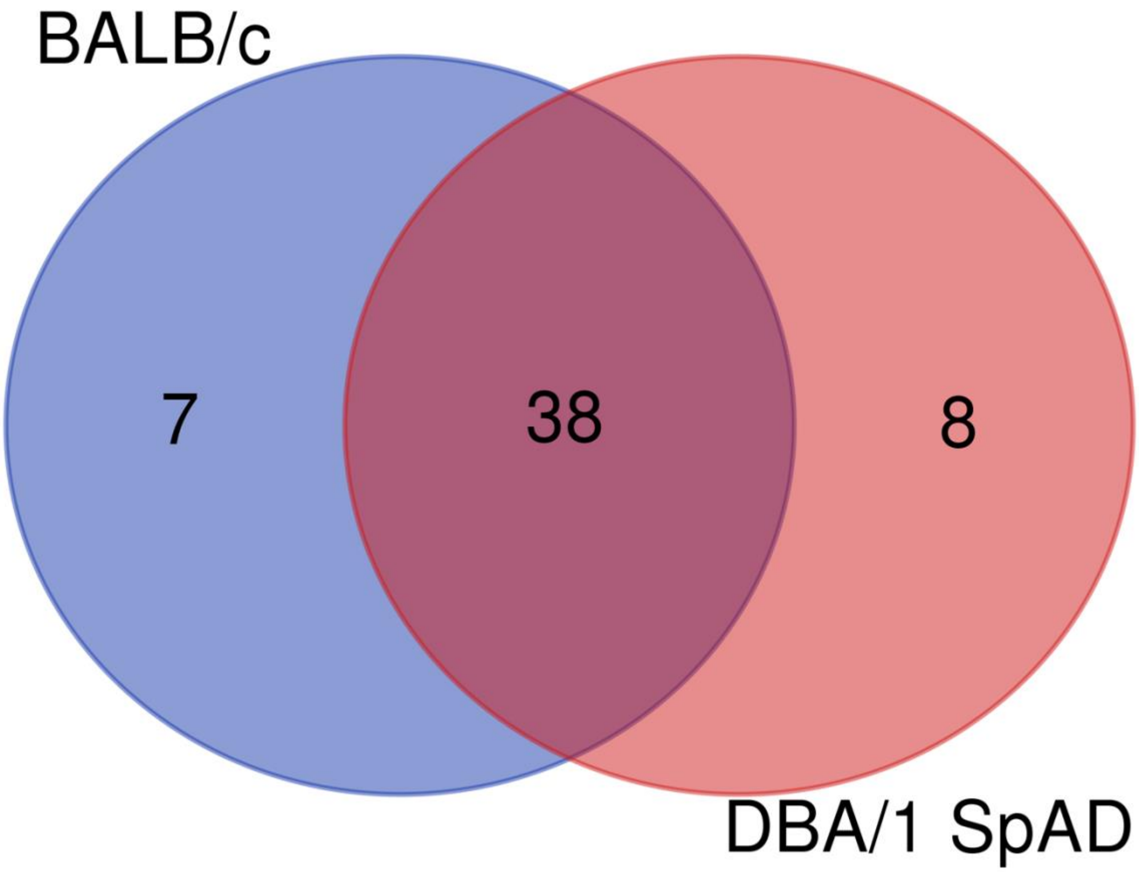

Group	Total	Bacterial species
BALB/c healthy<br><br>DBA/1 SpAD	38	Muribaculum_intestinale Paramuribaculum_intestinale Alistipes_A_871400_sp002362235 Odoribacter_splanchnicus Bacteroides_H_acidifaciens UBA3282_sp003611805 Eisenbergiella_tayi Helicobacter_D_mesocricetorum Prevotella_sp902776665 Phocaeicola_A_858004_sartorii Bacteroides_H_caecimuris Enterenecus_sp004560375 1XD8_76_sp003611955 G11_sp900103495 Mailhella_massiliensis Clostridium_Q_135822_fessum Bacteroides_H_rodentium CAG_269_sp000431335 Not_Assigned Parasutterella_sp009767915 Alistipes_A_871400_shahii Alloprevotella_sp002933955 Dysosmobacter_sp000403435 Paramuribaculum_sp001689565 Angelakisella_massiliensis Phocaeicola_A_858004_vulgatus CAG_485_sp002362485 Lactobacillus_johnsonii UBA3263_sp001689615 Lactobacillus_intestinalis Acetatifactor_sp011959105 Helicobacter_C_479931_typhlonius Lawsonibacter_sp014287875 Ventrimonas_sp003611875 BX12_sp902363595 Nanosyncoccus_sp003979185 Prevotella_sp004792655 Lawsonibacter_butyricus
BALB/c healthy	7	CAG_873_sp011959565 Emergencia_sp009935805 Muribaculum_gordoncarteri Dwaynesavagella_sp000270205 Evtepia_viridis UBA7173_sp002491305 Lawsonibacter_sp000177015
DBA/1 SpAD	8	Limosilactobacillus_reuteri Streptococcus_danieliae COE1_sp000403335 C_19_sp009917405 Mediterraneibacter_gnavus 1XD42_69_sp003612565 Faecalimonas_umbilicata 14_2_sp000403315
